# Accurate prediction of mega-electron-volt electron beam properties from UED using machine learning

**DOI:** 10.1038/s41598-021-93341-2

**Published:** 2021-07-06

**Authors:** Zhe Zhang, Xi Yang, Xiaobiao Huang, Junjie Li, Timur Shaftan, Victor Smaluk, Minghao Song, Weishi Wan, Lijun Wu, Yimei Zhu

**Affiliations:** 1 SLAC National Accelerator Laboratory, Menlo Park, CA 94025 USA; 2 National Synchrotron Light Source II, Brookhaven National Laboratory, Upton, NY 11973 USA; 3 Advanced Photon Source, Argonne National Laboratory, Lemont, IL 60439 USA; 4 Condensed Matter Physics and Materials Science Division, Brookhaven National Laboratory, Upton, NY 11973 USA; 5 School of Physical Science and Technology, ShanghaiTech University, Shanghai, 201210 China

**Keywords:** Condensed-matter physics, Experimental particle physics

## Abstract

To harness the full potential of the ultrafast electron diffraction (UED) and microscopy (UEM), we must know accurately the electron beam properties, such as emittance, energy spread, spatial-pointing jitter, and shot-to-shot energy fluctuation. Owing to the inherent fluctuations in UED/UEM instruments, obtaining such detailed knowledge requires real-time characterization of the beam properties for each electron bunch. While diagnostics of these properties exist, they are often invasive, and many of them cannot operate at a high repetition rate. Here, we present a technique to overcome such limitations. Employing a machine learning (ML) strategy, we can accurately predict electron beam properties for every shot using only parameters that are easily recorded at high repetition rate by the detector while the experiments are ongoing, by training a model on a small set of fully diagnosed bunches. Applying ML as real-time noninvasive diagnostics could enable some new capabilities, e.g., online optimization of the long-term stability and fine single-shot quality of the electron beam, filtering the events and making online corrections of the data for time-resolved UED, otherwise impossible. This opens the possibility of fully realizing the potential of high repetition rate UED and UEM for life science and condensed matter physics applications.

## Introduction

Mega-electron-volt (MeV) UED and UEM provide a unique opportunity of simultaneously high temporal and spatial resolution for time-resolved observations and measurements in physics, chemistry, and biology^1–12^. Examples include 3D-imaging thick biological samples such as cells in their native states and real-time visualizing structural dynamics in real space and in nanometer scales, which allows direct probing of charge-spin–lattice interactions to study the role of symmetry, topology, dimensionality, and correlations that control the exotic properties of quantum materials^13,14^. By employing an accelerator-based radiofrequency (RF) photoinjector as the MeV electron source, UED/UEM takes advantage of the strong interaction between electrons and matter while mitigating the space charge problem. To meet the long-standing scientific challenge of real-space imaging with a high spatiotemporal resolution of tens of nm × ps, packing a sufficient number of MeV electrons in the beam for time-resolved single-shot measurements in the presence of space charge is the key to success. Furthermore, due to the hundreds-times shorter wavelength compared to X-rays, electrons allow access to high scattering vectors in momentum space, so UED/UEM can potentially resolve much finer structural details. This is a big step towards a future possibility to see how protein–protein and protein-cell interact and to make molecular movies of chemical reactions. Together with XFEL these ultrafast probes will provide a more complete picture in groundbreaking studies of all kinds of complex dynamic processes in nature^5^.

Large energy spread and shot-to-shot spatial-pointing and energy jitters of the photocathode-based electron source are on the top of the list of technical challenges impeding MeV UED and UEM to reach their full potential as important tools in ultrafast science and technology. For example, numerical simulations carried out with the GPT code^15^ taking the stochastic-scattering and space-charge effects into full consideration show a significant increase in the energy spread of electron beam, from $1.5 \times 10^{{ - 3}}$ to $2.0 \times 10^{{ - 2}}$ when the beam charge increases from 1 to 16 pC. In the single-shot mode, the beam energy spread and normalized angular divergence are the main factors determining the diffraction peak width^10^. In the accumulation mode, the shot-to-shot energy fluctuation and spatial-pointing jitter could significantly increase the effective energy spread, which is the convolution of the single-shot energy spread and shot-to-shot energy jitter, and effective angular divergence, which is the convolution of the single-shot angular divergence and shot-to-shot spatial pointing jitter. A possible way around such fluctuations is to perform the full characterization of each electron bunch and use the real-time information to stabilize the machine and to retain only the events satisfying certain properties of the electron bunch. Moreover, by sorting the events and making online corrections of the data based on those characterizations, the jitter can be even utilized as an effective scan of the beam energy, energy spread, angular divergence, or spatial pointing^16–20^. The real-time nondestructive measurements of the energy spread and angular divergence could open the possibility of online optimization of the electron bunch at the sample, with the increased beam charge. This optimization could be crucial for the future development of a real single-shot time-resolved UED/UEM instrument^21,22^.

We have developed a diagnostic method based on Bragg Diffraction (BD). This method allows us to optimize the beam properties including energy spread, angular divergence, shot-to-shot pulse fluctuation and jitter, while the experiments are ongoing^19,20^. However, applying the BD diagnostic in real time is currently impossible for every electron pulse because the noisy background and instrument instability frequently overload the data stream and demand more complex numerical algorithms for the data analysis. In this paper, we propose ML as a general technique to overcome this limitation. Similar approaches have been successfully realized for a few scientific applications^23–28^. We demonstrate that ML can be applied at any UED/UEM facility to obtain the full electron bunch information with high fidelity on every shot even at the high repetition rate. Employing ML as a real-time noninvasive diagnostic could enable new features and capabilities, which are impossible for the present UED instruments. As an example, by adding a second set of the sample and detector pair downstream of the UED detector, those non-interacted electrons, originally being sent to the beam dump, will be diffracted to form a second BD pattern, as the input to the BD-based ML model. The electron beam properties predicted by the ML model could potentially be applied to automate the setup of the UED instrument and to perform the real-time data correction during the experiment. It is far beyond a diagnostic method of accelerators.

Using the data from simulation as a proof-of-principle, we show that much of the information including the energy, energy spread, spatial pointing and angular divergence of the electron bunches, which is usually extracted from complex invasive diagnostics such as dipole spectrometer and quadrupole based emittance scan^29^, is strongly correlated to the BD patterns recorded by the detector^20,21^. While these correlations are driven by physical processes, nevertheless performing accurate direct modeling of every experimental aspect in the machines as jittering as the UED/UEM is currently time-consuming and impossible in real time^20,21^. As an alternative, we use generic linear, quadratic and more complex but well-known ML models^30^, such as convolutional neural networks (CNN)^31,32^ or support vector regression^33^ to describe the non-trivial hidden correlations and make predictions of the fluctuations in the variables using the BD patterns as input. Those variables only can be measured by the complex destructive diagnostics before. We demonstrate the horizontal (*x*) and vertical (*y*) spatial pointing, *x* and *y* angular divergence, energy and energy spread prediction with the generalization errors around 0.01 regarding the normalized training variables. Furthermore, in the experiment at the BNL ATF, we show that similar predictability of *x* and *y* spatial pointing and energy jitters can be achieved with the RMS error of 0.11 μrad, 0.12 μrad, and 3.44 $\times 10^{{ - 5}}$, respectively, only using a small set (several thousand) of the BD patterns. This approach can be potentially applied at UED/UEM with a high repetition rate to provide accurate knowledge of complex electron bunches at the full repetition rate in real-time, as well as to lessen the load on the data stream requirement.

## Results

### Simulating BD patterns

We have completed the start-to-end simulation. An electron bunch emitted from the photocathode RF gun has been tracked through the UED beamline to the sample via the GPT code^15^. Afterwards, the electron diffraction is dynamically calculated using our own computer code, named electron diffraction patterns (EDP), for a single crystal. The code is based on the Bloch wave method, which takes dynamical effects in electron diffraction into a full consideration and has been successfully used for quantitatively determining crystal structure and charge distributions of crystals^34–36^.

The interface between the GPT and EDP simulation codes implemented in our early studies will be applied in calculating the electron diffraction patterns^21^. In the GPT code, each electron is defined as a particle, while a macroparticle represents a collection of electrons with the same properties, e.g. the coordinate in 6D phase space. The number of electrons in each macroparticle can be different. It is several thousand in our case. An electron beam can be therefore considered as a collection of macroparticles. With the macroparticle concept, a seamless transition between the GPT particle tracking from the gun to the sample and the EDP simulation of a wave-like electron being diffracted by sample, can be established. The electron beam incoming to the sample is represented by a collection of macroparticles in GPT simulation^15,36^. Each macroparticle has the coordinate $nmacro_{i}$, where *i* = 1, 2 to *N*, and *N* is the total number of macroparticles in the beam. *N* is equal to two thousand in our simulation. The beam charge is determined as $C = \mathop \sum \limits_{{i = 1}}^{N} c_{i} = \mathop \sum \limits_{{i = 1}}^{N} nmacro_{i} \cdot e$, where *e* is the charge of an electron. The reflection intensities are calculated based on the Bloch wave method for each individual macroparticle^21,34–36^ and the reflection intensities are scaled by the charge (number of electrons) of each macroparticle. The diffraction pattern is obtained by summation of reflection intensities from all macroparticles.

### ML with simulated BD patterns

#### A complete set of six training variables

To turn the ML model into a real-time non-destructive diagnostic tool, the choice of the training variables becomes critically important. We must choose the training variables such that they are not only predictable by the ML model with high accuracy but also easily projectable to the electron beam properties. This requires that the BD pattern as input to the ML model must contain adequate information of the desired electron beam properties. Since the spatial pointing (*θ*_*xc*_ and *θ*_*yc*_), angular divergence (∆*θ*_*x*_ and ∆*θ*_*y*_), energy (*E*) and energy spread (∆*E*/*E*) of the electron beam are intrinsically correlated to the BD peak widths and positions, these variables are ideal for the ML, based on these selection criteria. Then we performed the simulations of the BD patterns on a SrTiO_3_ single crystal sample using the EDP code. GPT simulation from the gun to the sample generates a realistic macroparticle distribution that contains the electron bunch energy, energy spread, angular divergence, and spatial pointing. The projection of the electron bunch properties to the training variables is analytically described by a linear function, as shown in Table 1. Using the macroparticle distribution as the input to the EDP simulation, the diffraction patterns are calculated as ‘tif’ format image files^34–36^. The diffraction intensity depends on the training variables *θ*_*xc*_, ∆*θ*_*x*_, *θ*_*yc*_, ∆*θ*_*y*_, ∆*E*/*E*, and *E*, as well as the information of the sample (e.g., thickness).

**Table 1:** The training variables *v*_1_, *v*_2_, *v*_3_, *v*_4_, *v*_5_, and *v*_6_ with respect to electron properties, *p*_1_, *p*_2_, *p*_3_, *p*_4_, *p*_5_ and *p*_6_, are listed, including the upper maximum and lower minimum limits for all parameters and the unit conversion between training variables to electron properties with their physical definitions.

Variable	Min	Max	Unit	Formula	Description	Variable in training	Normalization
*p*_*1*_	0	2.13 × 10^−4^	rad	∆*θ*_x_*	x-divergence	*v*_*1*_	*v*_*1*_ = *p*_*1*_/2.13 × 10^−4^
*p*_*2*_	− 1.73 × 10^−5^	1.73 × 10^−5^	rad	*θ*_*xc*_	x-jitter	*v*_*2*_	*v*_*2*_ = *p*_*2*_/3.46 × 10^−5^ + 0.5
*p*_*3*_	0	2.13 × 10^−4^	rad	∆*θ*_y_*	y-divergence	*v*_*3*_	*v*_*3*_ = *p*_*3*_/2.13 × 10^−4^
*p*_*4*_	− 1.73 × 10^−5^	1.73 × 10^−5^	rad	*θ*_*yc*_	y-jitter	*v*_*4*_	*v*_*4*_ = *p*_*4*_/3.46 × 10^−5^ + 0.5
*p*_*5*_	0	0.0013		∆*E*/*E*	energy spread	*v*_*5*_	*v*_*5*_ = *p*_*5*_/0.0013
*p*_*6*_	− 0.004	0.004		∆*E*_*c*_/*E*_*c*_	energy jitter	*v*_*6*_	*v*_*6*_ = *p*_*6*_/0.008 + 0.5

* When we speak about the divergence as the electron beam property, it means $\Delta \theta {\text{x}},{\text{y}}$; when we talk about the peak broadening, the divergence should be converted to the normalized divergence ∆*θ*_*x,y*_/*θ*_*x,y*_.

To generate the training dataset, those six parameters are varied as the input to the EDP simulation while all other parameters, e.g., the illumination area and the sample thickness, are fixed. The ranges of the variations are determined by the experimental setup of the UED facility at the BNL ATF^19,20^, as shown in Table 1. Special attention should be paid when we talk about the divergence in the training variables as well as in the EDP simulation, it means $\Delta \theta {\text{x}},{\text{y}}$; however, when we speak about the divergence induced BD peak broadening, it specifically indicates the normalized divergence $\Delta \theta {\text{x}},{\text{y}}/\theta {\text{x}},{\text{y}}$. Here $\theta {\text{x}},{\text{y}}^{*}$ is the BD angle of a particular BD peak in *x* or *y* direction, determined by the Miller index of the sample and the beam energy, usually in the range of sub-mrad to a few mrad.

We aim to build an ML model as the real-time UED/UEM diagnostic method. The ML model could become a virtual diagnostic tool of the electron bunch properties at the sample in real-time with the BD pattern on the detector as the input. A set of ten thousand images (simulated dataset #1) with the size of $256~ \times ~256$ pixels are generated based on the initial population set by the Latin hypercube sampling^37^. The Latin hypercube sampling method provides an efficient coverage of the parameter space with the maximum randomness and the highest density of the population. The labels of those images are normalized to the range of zero to one for the purpose of standardizing the training process. Since CNN is quite effective in the pattern recognition, it becomes the ideal ML model with the advantage of being robust and highly efficient in handling the sophisticated noisy background in the UED experiments. This statement can be justified by applying a similar ML method to the experimental dataset (described later in the manuscript).

**Figure 1:**
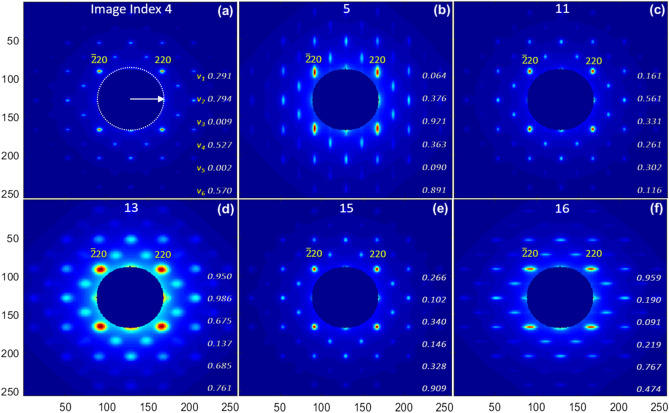
Simulated BD patterns of SrTiO_3_ single crystal along [001] direction with the index 4 (**a**), 5 (**b**), 11 (**c**), 13 (**d**), 15 (**e**) and 16 (**f**), from left-to-right and top-to-bottom. The corresponding training variables are listed as the data array on the right side of a BD pattern. Each image has the size of 65,536 (= 256 × 256) pixels, with the angular resolution of 20 pixel/mrad in the EDP simulation. For each image, the *x* and *y* view angles are the same, ± 6.40 mrad. Miller indexes of selected Bragg peaks, ($\bar{2}20$) and (220), are labeled (yellow). The cutoff radius, shown as the white dashed circle in the middle of Fig. 1a, represents the hole in the middle of the mirror which is part of the detector system. The detector includes a phosphor screen and a downstream mirror with a hole in the middle. The hole allows the background noises generated by the core of the non-interacted electron beam and by the dark current to pass through the mirror to the beam dump.

Examples of the input to the ML model, six BD patterns with the index of 4, 5, 11, 13, 15 and 16 in the simulated dataset are shown as Fig. 1a–f with the corresponding training variables listed as the inserts.

An ML model can be considered as a fitting function with the required complexities for reproducing the behavior of the experimental system. In our case, the experimental data are a series of the BD patterns recorded by the detector when the electron beam properties (spatial pointing, angular divergence, energy, and energy spread) are varied. Our goal is to find a fitting function that can map every BD pattern to its underlying electron beam properties. Since those BD patterns are in a high-dimensional space (65536-D), the fitting function must have enough degrees of freedom to account for the complexities of the correlations. Therefore, we choose a neural network (NN) based ML model in this study. The NN model^38^ typically consists of multiple layers of nodes, and the nodes between adjacent layers are connected through weighted non-linear functions, which are referred to as the activation functions. The weights in the NN model are determined by fitting the existing labeled data (in our case, the labels are the underlying electron beam properties that correspond to the BD patterns). This is the model training process. Typically, the existing data are divided into three subsets: the training set, the validation set, and the test set. The training and validation sets are used to fit the model and to prevent the model from overfitting, while the test set is used to check the accuracy of the trained model.

**Figure 2:**
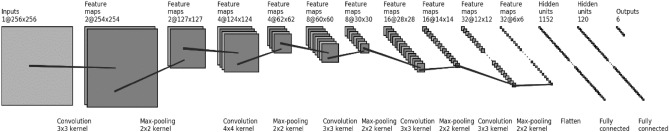
Schematic plot of the NN of the ML model including convolutional layer, pooling layer, and fully connected layer.

**Figure 3:**
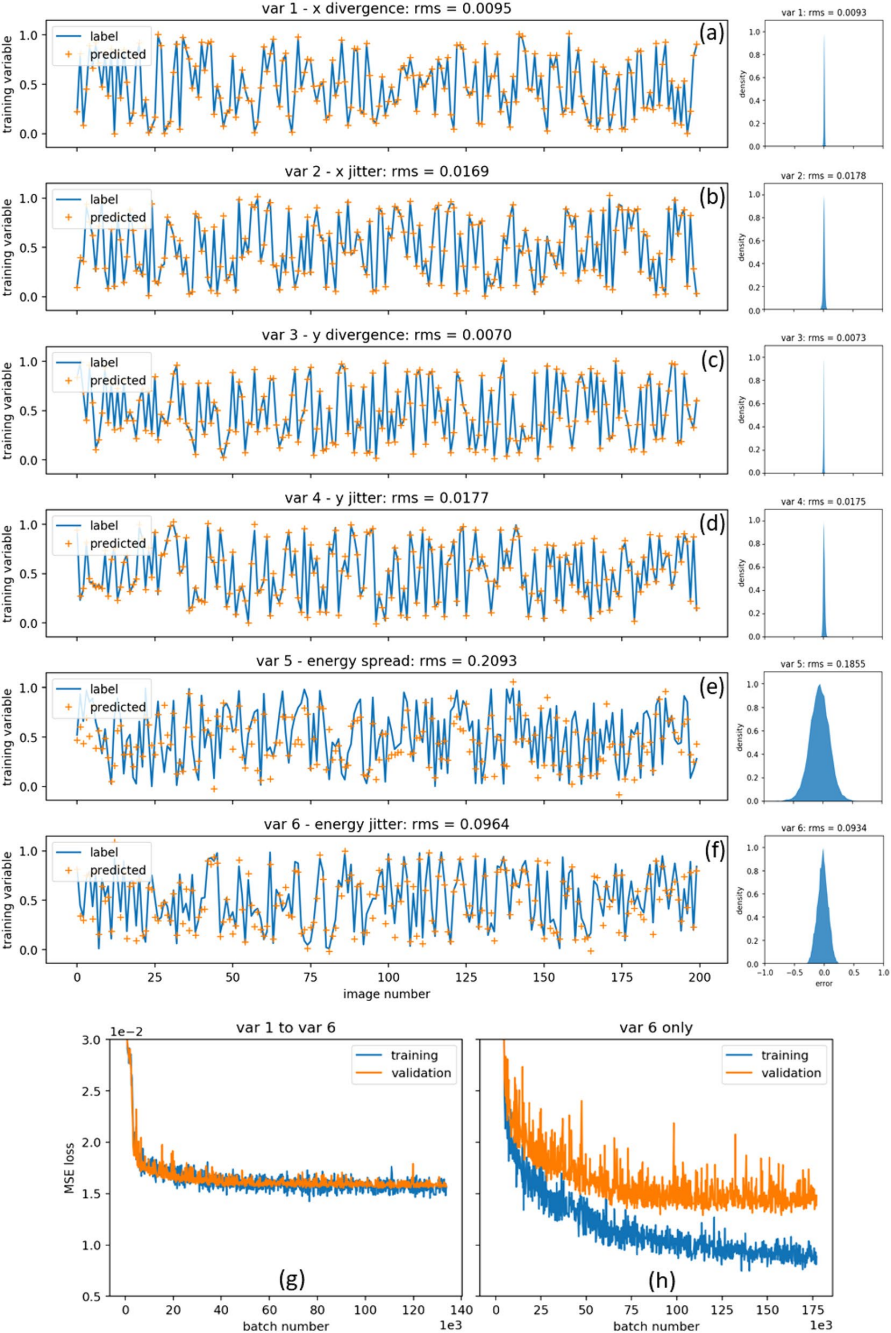
The training variables 1 (**a**), 2 (**b**), 3 (**c**), 4 (**d**), 5 (**e**), and 6 (**f**) as input to generate the BD patterns are plotted as the blue curve while their corresponding predictions by the ML model are plotted as the orange crosses for the untrained 200 images. Besides, the corresponding error distributions for the entire dataset of 10,000 images are plotted on the right side. The RMS errors for the untrained images and the entire dataset agree well. (**g**), (**h**): training loss (blue curve) and validation loss (orange curve) are plotted for two different cases, six training variables (*v*1 to *v*6) trained together and only variable *v*6 being trained, respectively. Notice the different *x* scaling for (g) and (h). The fluctuation of the loss curves for the variable *v*6 only is larger than the 6 variables due to the smoothing effect introduced by averaging the mean squared error (MSE) loss over all the variables in the 6-variable case.

The BD patterns are split into the three subsets with a ratio of 0.7:0.15:0.15. We choose a LeNet-style neural network architecture^39^ with a linear output activation function to meet the continuous output variable requirement, the actual network structure is schematically shown in Fig. 2. After a comprehensive training hyper-parameter tuning, including the image cropping size and the cutoff radius, with the help of the early stopping technique^40^ to reduce the training time and overfitting, the LeNet NN model can describe the non-trivial hidden correlations and make predictions of the fluctuations in the variables corresponding to the electron beam properties using the untrained BD patterns with a reasonably good agreement. The labels and the predictions of 200 images from the test set are plotted in Fig. 3a–f, as the blue curves and orange crosses, respectively, and show reasonably good agreements except variable 5 (*∆E*/*E*). Also, their corresponding error distributions regarding the whole image set are shown on the right side of Fig. 3a–f. Training loss (blue curve) and validation loss (orange curve) are plotted for two different cases, six training variables (*v*1 to *v*6) trained together and only variable *v*6 being trained, as Fig. 3g,h, respectively. It is clear to see that the generalization error of the prediction of variable 5 is about 10 times larger than the errors of variables 1—4, and 2 times larger than the error of variable 6. The generalization error is consistent with the RMS error of the randomly picked test images.

However, training variable 5 corresponding to the beam energy spread is the most challenging parameter to be trained with a predictable result. The initial training was unsuccessful because the BD peak widths in every image are dominated by the normalized angular divergence, not by the energy spread in the EDP simulation. The BD peak width is the convolution of the normalized angular divergence $\Delta \theta /\theta$ and the energy spread $\Delta E/E$: $\sqrt {\left( {\Delta \theta /\theta } \right)^{2} + \left( {\Delta E/E} \right)^{2} }$. In the first simulated dataset, the normalized angular divergence of $6.65 \times 10^{{ - 2}} \approx 2.13 \times 10^{{ - 4}} /3.2 \times 10^{{ - 3}}$ (the ratio of the angular divergence to the detector half view angle) is significantly larger than the energy spread of $1.3 \times 10^{{ - 3}}$ therefore, making the energy spread no longer detectable by the ML model.

#### Beam energy spread only

**Figure 4:**
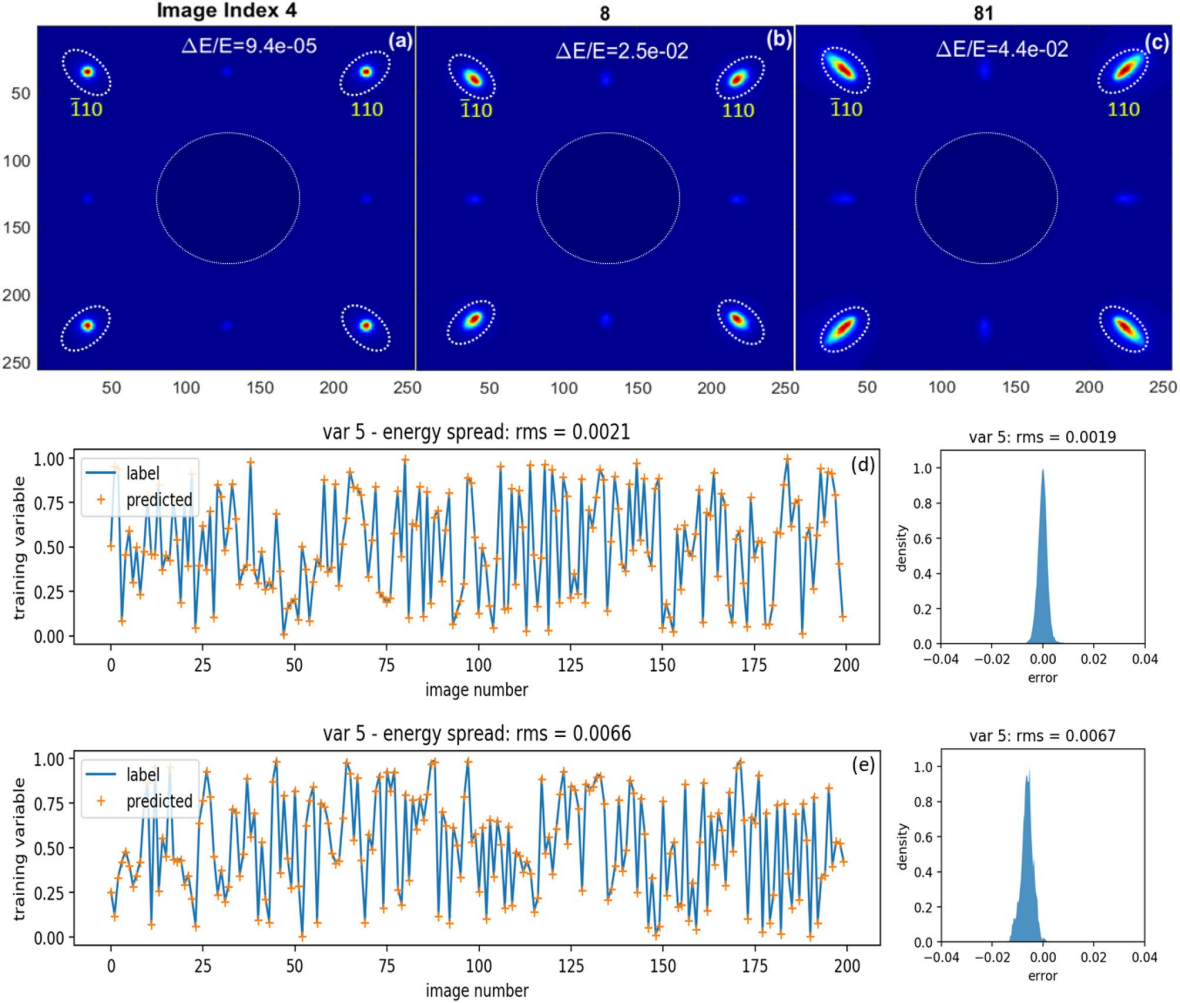
Simulated BD patterns of SrTiO_3_ single crystal along [001] direction of the dataset #2 with the RMS energy spread of 2 × 10^–2^ have the index 4 (**a**), 8 (**b**) and 81 (**c**) from left-to-right. Their corresponding training variables are 0.00204, 0.55412 and 0.95690, respectively. With the unit conversion of 0.04584, the energy spread is labeled in each image. Here, variable 5 multiplied by the unit conversion equals to the energy spread. The image has a size of 256 × 256 pixels, with an angular resolution of 100 pix/mrad in the EDP simulation. For each image, the view angles are the same in *x* and *y* directions: ± 1.28 mrad. Miller indexes of selected Bragg peaks, ($\bar{1}10$) and (110), are labeled (yellow). The dashed circle represents the hole of the mirror in the detector system. (**d**) The input data label is plotted as the blue curve while the corresponding prediction is plotted as the orange cross. The corresponding error distribution for the entire dataset of ten thousand images is plotted on the right side. (**e**) For the simulated dataset #3, with the RMS energy spread of 5 × 10^–3^, the input data label is plotted as the blue curve while the corresponding prediction is plotted as the orange cross. Similarly, the corresponding error distribution for the entire dataset of ten thousand images is plotted on the right side. The RMS errors for the untrained images and the entire dataset agree extremely well. The neural network architecture used for these trainings is almost identical to the one shown in Fig. 2, with the only difference as the number of variables in the output layer.

For the proof-of-principle purpose, we only vary the variable 5 in the EDP simulation while keeping all other training variables fixed (named simulated dataset #2). To make the BD peak width dominated by the beam energy spread, we deliberately choose the maximum energy spread (2.0% RMS) for the high-charge UED experiments while keeping the *x* and *y* divergences within a few microradian in RMS. In the current UED at BNL, the angular divergence at the sample can be varied in a limited tuning range using a solenoid as the only knob. Assuming the geometrical emittance of the electron beam is 10^–8^ m rad, and the virtual image is 100 μm in diameter, the beam divergence at the sample can vary from 97 μrad to 677 μrad. Additional condenser lenses can be implemented together with an aperture to further reduce the angular divergence (see the details in “Discussion” section). By doing so, we generate the second set of 10,000 simulated BD patterns with the peak widths dominated by the energy spread. Also, there is a clear feature difference between the peak broadening induced by the angular divergence and the broadening induced by the energy spread. The energy spread causes the peak broadening in the radial direction, as shown in Fig. 4a–c; whereas the *x* and *y* angular divergences broaden the BD peak in the same direction with the beam divergence.

There is such a striking feature that reflects the correlation between the energy spread and BD peak broadening in the second dataset of simulated BD patterns, as one can see in Fig. 4a–c, dashed ellipses on each image. So, we can achieve similar predictability of the beam energy spread with the mean error of 0.002, as shown in Fig. 4d, which is converted to excellent accuracy of the energy spread prediction with an error of 9.2 × 10^–5^.

To explore the ultimate predictability of the electron beam energy spread by an ML model, a new dataset #3 of 10,000 BD patterns have been generated, with the only difference of a four times smaller energy spread of 5 × 10^–3^ compared to the dataset #2. The training result is shown in Fig. 4e. As one expects, a similar precision with minor degradation has been achieved with the mean error of 0.0066 with respect to those randomly selected two hundred untrained images.

So far, we have successfully demonstrated for the two major sources that the angular divergence often contributes the most significant part of the peak broadening compared to the energy spread. As an example, the angular divergence, Bragg angle with the first order of reflection, and energy spread are chosen to be 0.2 mrad, 1.0 mrad and 0.01; the peak broadening due to the angular divergence and the energy spread are 95% and 5%, respectively. Making the prediction of energy spread with the agreement better than 10% has been proved to be quite a challenge not possible in the current ATF setup. This is evidenced by the simulated case #1. If we choose the Bragg angle with the third order of reflection, those peak broadenings become 87% and 13%, respectively. However, this requires some significant improvements to the detector system. Instead, to make the ML model with the required predictability of the energy spread, we propose a novel approach via experimentally creating a similar condition of the simulated cases #2 and #3, see details in “Discussion”.

### ML with experimental BD patterns

We applied the ML to the diffraction patterns of the MoTe_2_ sample taken at the BNL UED in 2020. To answer the elusive question, whether all the six training variables are suitable for the ML in the experimental case, we must decide which parameters can be trained effectively in the ML model with the required precision. From our past experience^20^, the conventional data analysis only can provide variable 2, 4 and 6 with high fidelity; it is not possible for variable 1, 3 and 5. The reason is that the information of variable 1, 3, 5 and variable 2, 4, 6 are intrinsically correlated to the BD peak widths and positions, respectively. The peak position can be fitted with a high accuracy of 10^–4^ using the standard Gaussian method^20^. However, it is difficult to achieve a similar 10^–4^ precision with respect to the fitted peak width, often worse than 10^–3^ in the UED experiments.

**Figure 5:**
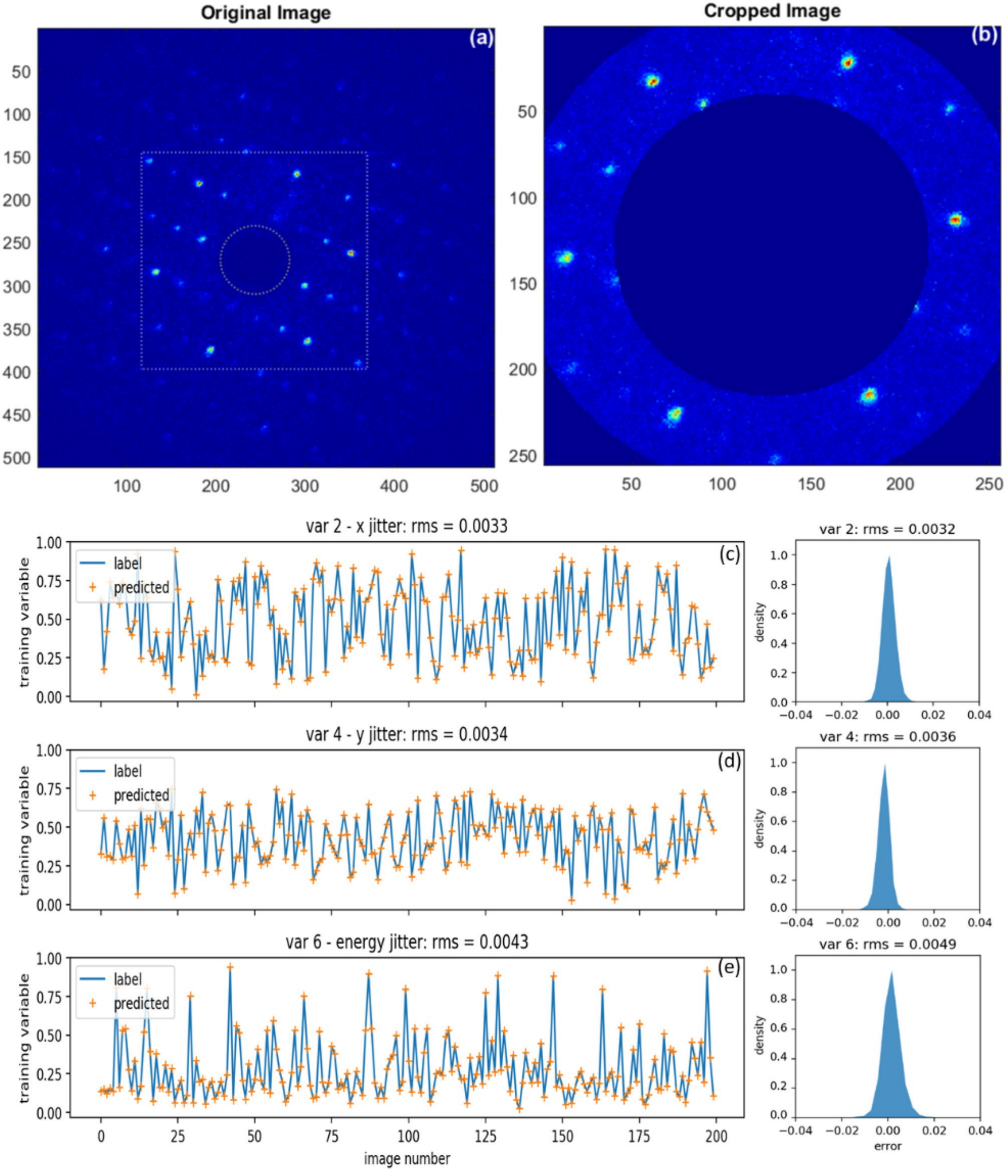
An example of original experimental image (**a**). The white box indicates the selected area for the data analysis. The dashed circle represents the hole of the mirror in the detector system. Cropped image for the ML (**b**). Training variables 2 (**c**), 4 (**d**), and 6 (**e**) via the data analysis using the Gaussian method are plotted as the blue curve while their corresponding predictions as the result of the ML model are plotted as the orange crosses respectively for the untrained two hundred images. Besides, their corresponding error distributions for the entire dataset of ten thousand images are plotted on the right side. The RMS errors for the untrained images and the entire dataset agree extremely well.

Furthermore, to differentiate the peak broadening due to the energy spread and angular divergence with a similar precision of 10^–4^, the diffraction pattern must contain the BD peaks higher than the first order of reflection as well as having an adequate resolution and reasonable signal to noise ratio (SNR) of the detector^20,21^. This is not possible in the current UED facility at the BNL ATF, which is limited by the view angle and resolution of the detector. Only the BD peaks with the first order of reflection are available, as shown in Fig. 5a indicated by the white dashed square.

We only can obtain the data label of each image for variable 2, 4, 6 via the Gaussian method^20^; therefore, the training must be limited to variable 2, 4, and 6 in the experimental case. The number of variables in the output layer of our ML model has been set to 3 instead of 6, to reflect this change. We achieve a similar predictability of *x* and *y* spatial pointing and energy jitters with the RMS error of 0.11 μrad, 0.12 μrad, and 3.44 $\times 10^{{ - 5}}$, respectively, as showing in Fig. 5c–e. Training loss and validation loss will be shown in “Pre-processing BD patterns towards a compact ML model”. To make the high-order BD peaks visible with the required SNR, we must increase the range of view angles and improve the detector resolution.

### ML as real-time UED diagnostic tool

**Figure 6:**
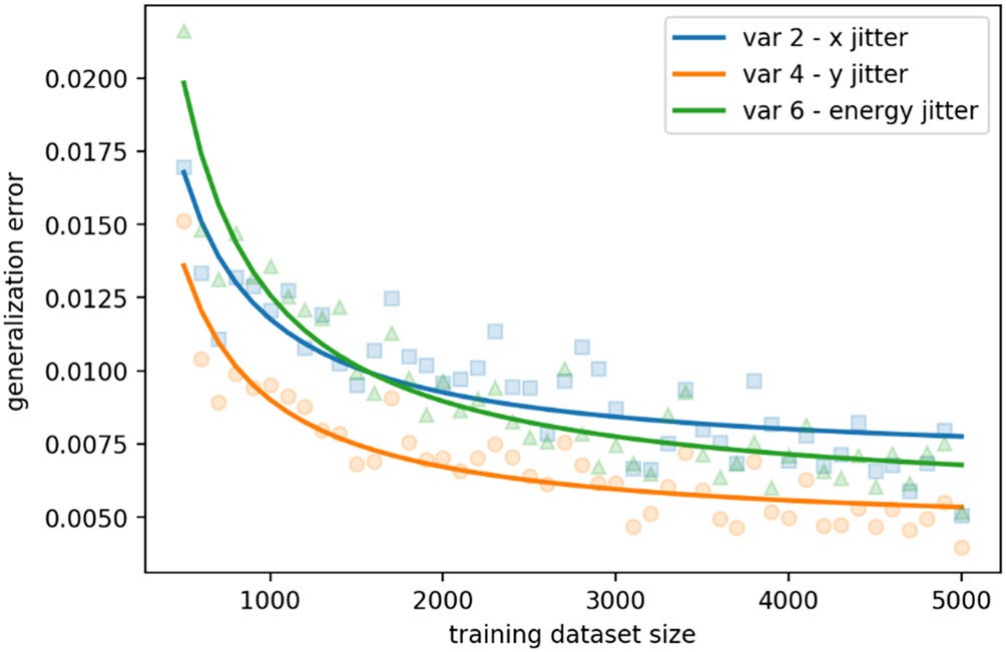
The results for one round of training with image numbers ranging from 500 to 5000 with a step size of 100. The generalization error in the plot is the RMS error of the trained model prediction based on all the available data of 5000 images. The scatter points are the raw data and the solid lines are the inverse fittings to the raw data^41^. The training for each dataset size has been rerun for 10 times to get less noisy data.

Since the sample needs to be changed very often for different experiments, it is important that by training an ML model on a small set of fully diagnosed electron bunches, we can accurately predict beam properties for every shot using only parameters that are recorded at the high repetition rate by the detector as the bonus while the experiments are ongoing. This could open the door to fully realizing the BD-based ML model as a real-time single-shot diagnostic tool widely applicable to different UED and UEM experiments. Whenever there is a sample replacement or a machine change, only several thousand BD patterns are needed for retraining and adapting the ML model to the new condition, to meet a 0.01 generalization error threshold, as shown in Fig. 6. For the *x* and *y* spatial pointing (blue square and orange circle) and energy (green triangle) jitters, only 3000 to 4000 images are needed for a predictable ML model with an accuracy better than 99%. It takes only about 20 min on a NVIDIA GeForce RTX 2080 GPU card in the experiment at the BNL ATF. With the accumulation of more data, taken at different electron beam properties and sample varieties, the model will continuously grow until it can cover most of the routine operations, therefore no need for retraining.

### Pre-processing BD patterns towards a compact ML model

**Figure 7:**
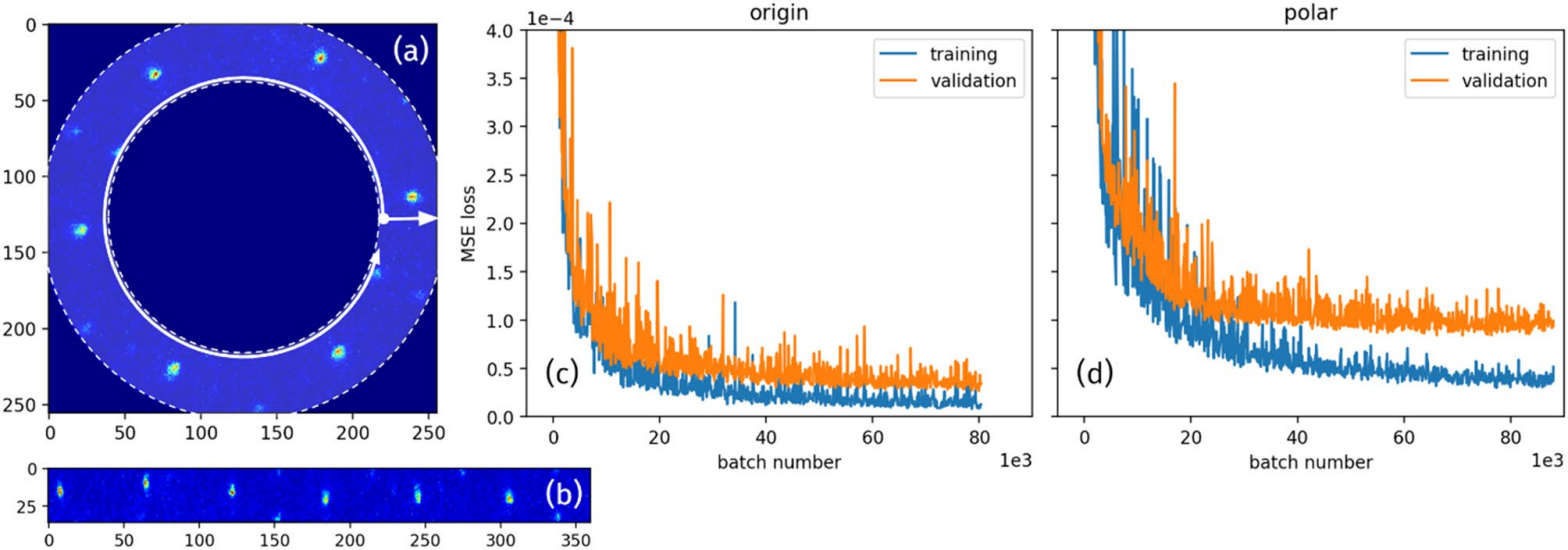
(**a**) An example of the original experimental image. The highlighted dashed ring area indicates the informative part of the image. The two arrows show the axes of the polar coordinate that the ring to be transformed to. (**b**) The polar-transformed image of the ring area in (**a**). (**c**), (**d**) Training loss (blue curves) and validation loss (orange curves) for the original images and the polar images, respectively.

To further reduce the training time, we explored the possibilities of using more compact BD patterns and a simpler ML model, with an acceptable trade-off on the prediction accuracy. Taking one experimental BD pattern shown in Fig. 7a as an example, useful information is carried mainly by the pixels within the ring. It would be a waste to employ a CNN that accepts the full 256 × 256 image as input. The number of informative pixels is approximately 38% of the total. If the image could be compressed in a way that only the pixels in the ring are kept, one can expect a significant reduction of the total number of the parameters of the model, speeding up the training and inference processes.

A polar coordinate transformation with resampling has been applied to extract the pixels in the ring into a rectangular image. The transformed input image of Fig. 7a is shown as Fig. 7b. By choosing a radial interval of [91, 127] pixel with 1 pixel tick and angular interval of [0, 360] degree with 1 degree tick, the size of the input image has been reduced by 80%, while the number of the model parameters reduced by 70%. With the compact input images and the model structure, the training time was shortened to around 8 min. Given the fact that for the original dataset the training takes around 20 min to converge, the time reduction is about 60%.

**Figure 8:**
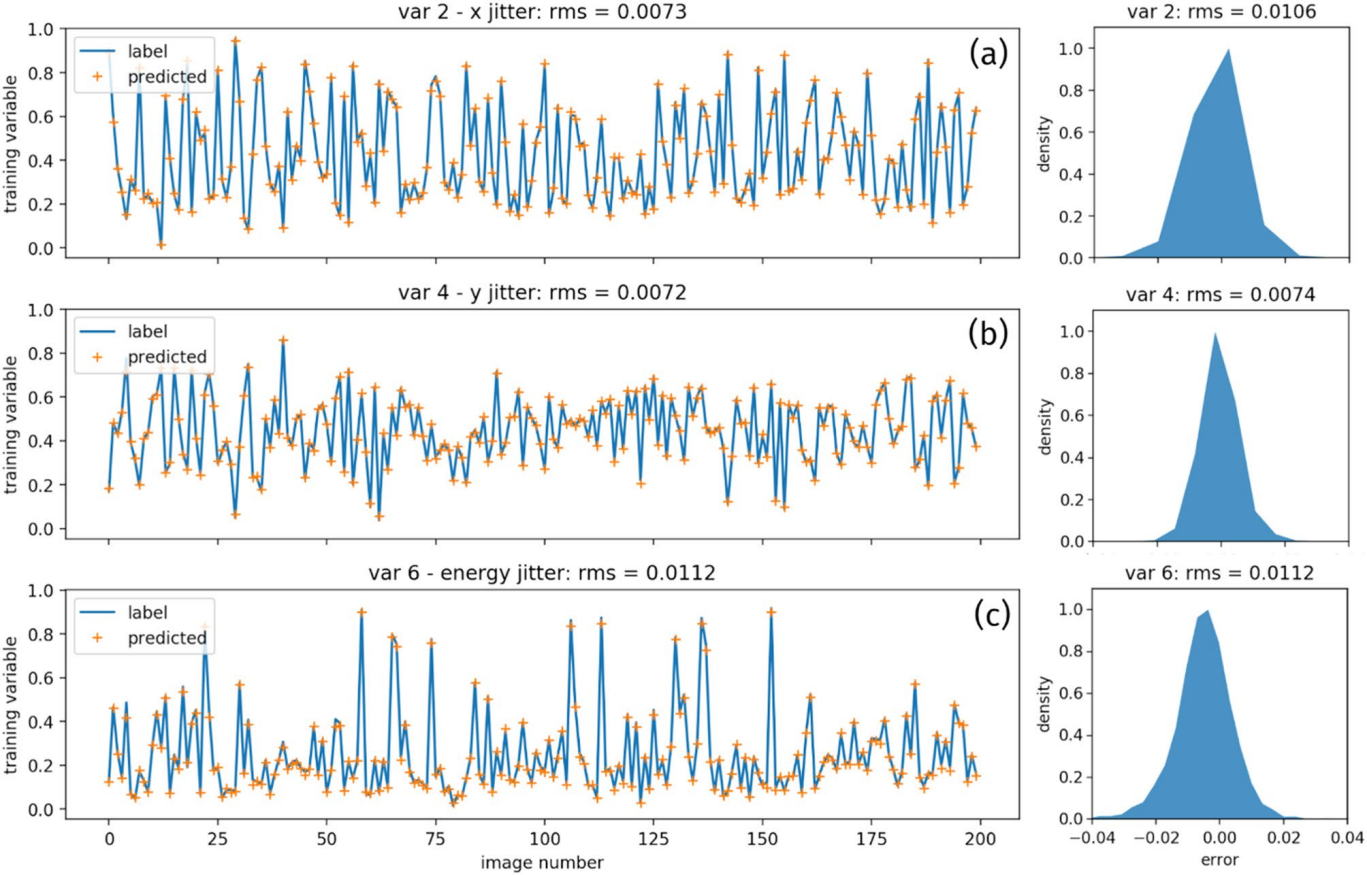
Training variables 2 (**a**), 4 (**b**), and 6 (**c**), labels are plotted as the blue curve while their corresponding predictions as the result of the polar ML model are plotted as the orange crosses respectively for the untrained two hundred images. Besides, their corresponding error distributions for the entire dataset of five thousand images are plotted on the right side. The RMS errors for the untrained images and the entire dataset agree extremely well.

The price of shortening the training time is the degradation of the prediction accuracy. As Figs. 7c,d, and 8a,c indicate, the predicted RMS error for *x* jitter, *y* jitter and energy jitter of the polar ML model are 0.0106, 0.0074 and 0.0112, respectively, which are almost twice as large compared to the ones of the original ML model for the experimental data, as shown in Fig. 5c–e. This degradation of performance is expected because the spatial structure of the spots in the ring region was lost during the polar transformation, while such information is crucial for the extraction of the spatial pointing and energy jitter. The compact CNN model must recover this spatial relationship to make more accurate predictions. While in practice, the depth of the neural network is usually not going to be set deep enough due to various reasons, such as reducing the training time for our case. Therefore, the model might not be able to easily learn this kind of high-level features, which leads to inferior performance.

The standard size of the detector of a cryo-electron microscope is 4000 × 4000. Only a small fraction (e.g. 10%) of those pixels contain useful information, depending on the specific sample setup. It is evident that the image size reduction and coordinate transformation could result in a much more compact and efficient ML model, enabling real-time event selection, data correction, as well as electron beam diagnostics.

## Methods

The start-to-end simulation is performed via the GPT particle tracking from the gun to the sample and the EDP simulation of a wave-like electron being diffracted by the sample. The BD patterns as input to the ML model are labeled by the electron beam properties used to generate those diffraction patterns. To meet the continuous output variable requirement, a LeNet^39^-style neural network architecture with a linear output activation function was used as the ML model.

The diffraction patterns of the MoTe_2_ sample taken at the BNL UED in 2020 were used as the input to the ML model in the experimental case. Based on our past experience^20^, the conventional data analysis via the Gaussian method can provide variable 2 (*x* jitter), 4 (*y* jitter) and 6 (energy jitter) with high fidelity since they are intrinsically correlated to the BD peak positions that can be fitted with the high accuracy of 10^–4^. The training was limited to variable 2, 4, and 6. The number of variables in the output layer of our ML model was set to 3 instead of 6, to reflect this change. To further reduce the training time, pre-processing BD patterns via image cropping and polar coordinate transformation was explored. A compact ML model has been obtained to speed up the training process by 60% with the cost of a minor reduction of the prediction accuracy.

## Discussion

To maintain the long-term stability as well as to achieve the fine single-shot quality of the electron beam, the constantly drifting UED/UEM makes the ML technique essential not only to provide a good condition for the machine startup but also to feed the real-time information of the electron bunch properties for online optimization.

We demonstrated the ML approach as the single-shot real-time diagnostics of the transverse electron beam properties. In the simulation case, the ML model can go beyond the transverse diagnostics even predicting the energy spread of an electron beam. However, there are some obstacles that we must overcome before the ML technique can be applied to extract the full information of the electron bunch including the energy spread. Since an ML model built on the simulated dataset #2 or #3 can predict the beam energy spread with the required precision, it is important that we can experimentally reproduce the simulation condition. Thanks to the independent control of the beam size and angular divergence via a set of condenser lenses, which can be either round lenses or a few quadrupole magnets^19^, a beam waist can be formed at the sample and the angular divergence can be adjusted freely. To maintain a reasonable illumination area, an aperture can be placed upstream of the sample. With this setup, the minimum angular divergence may be as small as a few μrad. In addition, the energy spread can be increased with the increase of the beam charge, as the result of increasing the photocathode drive laser power^21^. This allows us to vary the upper limit of the beam energy spread in a broader range for the training dataset, toward the required measurement precision of better than 10^–3^.

To be specific, the required measurement precision is similar for both the conventional BD method and the BD-based ML method. However, the ML method is much faster because the data collection and model training are done before the model is used. Evaluation of the CNN model with each BDP to predict the electron beam properties takes only 1.65 ms using 4.4 GHz CPU and 0.39 ms using RTX2080 GPU in the single mode, instead of 300 ms or more required by the conventional method using the available 4.4 GHz CPU. For a UED operated at a repetition rate above 3 Hz, the BD-based ML method is significantly superior because it is faster by several orders of magnitude. If thousands of frames are collected and processed all at once in the batch mode, the speed gain is even larger, as shown in Table 2.

**Table 2:** The speed test is done in both the single mode and the batch mode.

	Conventional BD method		BD-based ML method	
	Time per frame (s)	Max. repetition rate (Hz)	Time per frame (s)	Max. repetition rate (Hz)
4.4 GHz CPU (single mode)	4.0 × 10^−1^	2.5	1.65 × 10^−3^	0.6 × 10^3^
RTX2080 GPU (single mode)			0.39 × 10^−3^	2.6 × 10^3^
4.4 GHz CPU (batch mode)	3.0 × 10^−1^	3.3	0.95 × 10^−3^	1.1 × 10^3^
RTX2080 GPU (batch mode)			0.50 × 10^−6^	2.0 × 10^6^

The batch mode is to apply the method to 5000 frames at once, while the single mode is to apply the method to one frame at a time.

Our next goal is to develop an integrated product, including the hardware and an ML model, as a single-shot noninvasive real-time beam diagnostic tool for future MeV UED and UEM facilities with a high repetition rate. The hardware will aim for a standalone diagnostic toolbox, including the sample for calibrating the ML model, alignment parts, viewport, vacuum connections, etc., while the software could be the ML model for online monitoring and tuning of the angular divergence, energy spread, shot-to-shot energy and spatial pointing jitters of the electron beam. This new diagnostic toolbox could be an important step forward for providing essential information of the electron beam in real-time, which would help to achieve the long-term stability and fine single-shot quality of the electron beam.

Furthermore, the BD-based ML method provides the real-time, single-shot characterization of the electron beam properties at a high repetition rate up to 2 kHz. The repetition rate can be increased to MHz level if the data are processed in the batch mode. This will potentially enable fully automated UED operation. To achieve this ultimate goal, our plan is to perform a feasibility study using GPT simulation from the gun to the sample. Then, a second ML model, the gun-to-sample ML model, will be built with the simulation data. The input to this ML model will be the electron beam properties and the output will be the machine parameters. When we apply the method to experiments, the electron beam properties will first be obtained from the prediction of the BD-based ML model; then, the gun-to-sample ML model will make the prediction of the machine parameters, which will, in turn, be used to set up the UED instrument. The two-stage ML approach has much broader applications due to the availability of the electron beam properties. One can apply this two-stage approach not only to automate the setup of the UED instrument but also to perform the real-time data correction using the available electron beam properties during the experiment. This will be investigated in future studies.
